# Poly(d,l-Lactic acid) Composite Foams Containing Phosphate Glass Particles Produced via Solid-State Foaming Using CO_2_ for Bone Tissue Engineering Applications

**DOI:** 10.3390/polym12010231

**Published:** 2020-01-17

**Authors:** Maziar Shah Mohammadi, Ehsan Rezabeigi, Jason Bertram, Benedetto Marelli, Richard Gendron, Showan N. Nazhat, Martin N. Bureau

**Affiliations:** 1 Department of Mining and Materials Engineering, McGill University, Montreal, QC H3A 0C5, Canada; Maziar.ShahMohammadi@mail.mcgill.ca (M.S.M.); Ehsan.Rezabeigi@mail.mcgill.ca (E.R.); Jason.Bertram@mail.mcgill.ca (J.B.); Benedetto.Marelli@mail.mcgill.ca (B.M.); mbureau@sanexen.com (M.N.B.); 2 National Research Council Canada, Boucherville, QC J4B 6Y4, Canada; rgendron_780@hotmail.com

**Keywords:** phosphate-based glass particulates, poly(d,l-lactic) acid, biodegradable composites, solid-state foaming, carbon dioxide, tissue engineering

## Abstract

This study reports on the production and characterization of highly porous (up to 91%) composite foams for potential bone tissue engineering (BTE) applications. A calcium phosphate-based glass particulate (PGP) filler of the formulation 50P_2_O_5_-40CaO-10TiO_2_ mol.%, was incorporated into biodegradable poly(d,l-lactic acid) (PDLLA) at 5, 10, 20, and 30 vol.%. The composites were fabricated by melt compounding (extrusion) and compression molding, and converted into porous structures through solid-state foaming (SSF) using high-pressure gaseous carbon dioxide. The morphological and mechanical properties of neat PDLLA and composites in both nonporous and porous states were examined. Scanning electron microscopy micrographs showed that the PGPs were well dispersed throughout the matrices. The highly porous composite systems exhibited improved compressive strength and Young’s modulus (up to >2-fold) and well-interconnected macropores (up to ~78% open pores at 30 vol.% PGP) compared to those of the neat PDLLA foam. The pore size of the composite foams decreased with increasing PGPs content from an average of 920 µm for neat PDLLA foam to 190 µm for PDLLA-30PGP. Furthermore, the experimental data was in line with the Gibson and Ashby model, and effective microstructural changes were confirmed to occur upon 30 vol.% PGP incorporation. Interestingly, the SSF technique allowed for a high incorporation of bioactive particles (up to 30 vol.%—equivalent to ~46 wt.%) while maintaining the morphological and mechanical criteria required for BTE scaffolds. Based on the results, the SSF technique can offer more advantages and flexibility for designing composite foams with tunable characteristics compared to other methods used for the fabrication of BTE scaffolds.

## 1. Introduction

Bone tissue engineering (BTE), which aims to use a highly porous scaffold construct to promote the regeneration of the damaged tissue, is a promising alternative to current surgical bone grafting techniques [1,2,3]. An ideal scaffold for BTE applications should have biodegradability, biocompatibility, interconnected porosity with appropriate pore size and mechanical properties comparable to those of the native tissues to be able to withstand the applied forces [4,5,6,7]. It has been shown that highly porous composites with a polymeric matrix containing bioactive particles can offer promising alternatives [8,9].

Attributable to their bioactivity, the use of bioceramics has been shown to improve the overall biological response of the scaffolds [10,11]. For example, calcium phosphate ceramics and glasses, such as hydroxyapatite (HA) and silicate-based glasses (e.g., Bioglass®) incorporated into biodegradable polymers, e.g., poly(lactic acid) (PLA), poly(glycolic acid) (PGA), and their copolymers (PLGA) [12], as well as polycaprolactone (PCL) [13] have been extensively studied for BTE applications [12,14,15]. Because of their controllable solubility in aqueous environments (e.g., physiological fluids), phosphate-based glasses (PGs) can be an alternative inorganic phase for the purpose of producing bioactive composites with controllable degradation characteristics [16,17,18].

The selection of the foaming technique has a critical effect on the in vivo performance of the scaffold. Thus, over the past few decades, many methods such as thermally or nonsolvent-induced phase separation [9,12,19,20], electrospinning [21,22], and 3D printing [23,24] have been used to produce bone scaffolds. In terms of scaffold production, batch foaming using atmospheric gases such as carbon dioxide (CO_2_) and nitrogen has been developed to generate highly porous foams, yet avoiding the use of either organic solvents or high temperatures [25]. CO_2_ is usually the preferred candidate, which can act as a porogen to create 3D polymeric cellular structures [26,27]. Batch foaming using CO_2_ is often performed above the critical temperature (*T*_c_ = 30.95 °C) and pressure (*P*_c_ = 7.38 MPa) of CO_2_; a process referred to as supercritical foaming [26,27]. For example, Georgiou et al. [28] produced and investigated PLA-(0–20 wt.%)PG (50P_2_O_5_-46CaO-4Na_2_O) composite foams for BTE by using supercritical CO_2_ (scCO_2_) (*T* = 195 °C and *P*_sat_ = 15–25 MPa). It was shown that smaller pores were created with increasing amounts of glass content. However, the foaming process was not efficient for higher filler content composites (e.g., PLA-20 wt.% PG) in terms of the amount of pore formation [28].

In the solid-state foaming (SSF) technique, which is a green, solvent-free foaming approach, the polymer sample is saturated with CO_2_ at room temperature in a high-pressure autoclave. The gas-charged sample is then removed from the autoclave while its solid state at room temperature prevents foaming. The polymer then expands after heating above its glass transition temperature (*T*_g_). This method is directly related to the plasticizing properties of CO_2_, where the dissolution of CO_2_ results in a reduction of the *T*_g_ of amorphous polymers because of the thermodynamic plasticization effect of CO_2_ in the intermolecular network of the polymer [20,25]. As the temperature is increased above the reduced *T*_g_, resulting from the remaining gas dissolved in the polymer, nucleation and growth of pores can occur. In order to maximize the efficiency of the dissolving gas, the final temperature is usually set above the *T*_g_ of the polymer. If the plasticization level is such that the resulting *T*_g_ is well below the room temperature, e.g., by using very high-pressure CO_2_, the foaming technique can be performed at room temperature during the depressurization step [20,25,28,29]. Mooney et al. [29] developed this technique by foaming poly(d,l-lactic-co-glycolic acid). The samples were exposed to high-pressure CO_2_ (5.5 MPa) for 72 hours at room temperature followed by a pressure drop to atmospheric levels. Highly porous structures (up to 93%) with pore sizes around 100 µm were produced via this technique [29]. Therefore, a potential advantage of SSF is the ability to achieve high porosity at greater filler content by optimizing the process parameters. In addition, this technique allows for lower processing temperatures, which would be beneficial in preserving the molecular weight of polymer matrix or in case of incorporation of therapeutic agents.

In this work, a SSF method was developed and used to sustainably produce highly porous poly(d,l-lactic acid) (PDLLA) containing higher volume fractions of PG particulates (PGPs) with appropriate mechanical properties and morphologies in terms of porosity, pore size, and interconnectivity for potential BTE applications; all of which combined may not be achieved via other commonly used foaming techniques.

## 2. Experimental

### 2.1. Composite and Foam Fabrication

Fully amorphous, biodegradable PDLLA (Purasorb^®^ PDL 05, Purac Biochem, Goerinchem, Netherlands) with a density of 1.26 g/cm^3^, an inherent viscosity of 1.58 dL/g, molecular weight (*M*_w_) of 60 kDa, and *T*_g_ of 65 °C was used as the matrix of the monoliths without further purification. Melt-derived PGPs with the composition of 50P_2_O_5_-40CaO-10TiO_2_ (mol.%), produced as previously described [30], with median d50 (i.e., the value of the particle diameter at 50% in the cumulative distribution) of 50 µm was used as the bioactive filler. This PG composition has been assessed in terms of its degradation, ion release, and cytocompatibility using MC3T3-E1 pre-osteoblasts, and showed cell attachment and viability with a confluent growth, as well as proliferation and alkaline phosphatase production [30].

PDLLA-PGP composites were melt-extruded using a micro-compounder (Thermo Fisher Scientific HAAKE MiniLab, Berlin, Germany) with two conical co-rotating screws of reduced capacity (5 cm^3^). Initially, PDLLA pellets were dry mixed alone or with 5, 10, 20, or 30 vol.% PGP and left overnight under vacuum at 45 °C. Melt-extrusion was then performed at 110 °C under a flow of nitrogen with a screw rotation speed of 100 rpm and an overall residence time of 4 min. The extruded composite rods were cut into small pieces (for homogeneity of the bulk composite) and were dried overnight under vacuum at 45 °C prior to compression molding. Compression molding was carried out in a closed mold using a Carver Laboratory Press (Model M, Carver Inc., Wabash, IN, USA) by applying 2 MPa at 160 °C for 10 min followed by cooling to room temperature. Table 1 presents the material codes and compositions.

Neat PDLLA and PDLLA-PGP composite foams were generated using SSF. The composite monoliths (10 × 10 × 2.6 mm^3^) were saturated with CO_2_ in an autoclave (E3000, Quorum Technologies Ltd., Lewes, UK) attached to a CO_2_ cylinder. Prior to foaming, the room temperature solubility of CO_2_ in PDLLA at different pressures and times was determined using initial slope method (extrapolation) after the sample was removed from the autoclave (Supplementary Table S1). This step enabled the determination of the adequate conditions and concentration of CO_2_. The CO_2_ pressure for SSF was finally set to 2.4 MPa and the samples were kept at this pressure for at least 3 days to ensure that the CO_2_ content was 5.48 wt.%. The specimens were then retrieved from the pressure vessel and brought to atmospheric pressure. Foaming was conducted through an abrupt temperature rise using an oven which was set at 80 °C. Exposure to heat for 1 to 2 min was sufficient for the samples to be foamed.

### 2.2. Composite and Foam Characterization

#### 2.2.1. Density and Molecular Weight (*M*_w_) of the Polymer Matrix after Composite Processing

Archimedes’ Principle in isopropanol (A416-20, Fisher Chemical, Ottawa, ON, Canada) was used to experimentally measure the density of the composites. Isopropanol was used instead of water as PGs are hydrolysable in aqueous environments. Equation (1) was used to calculate the density of the composites.

(1) $$\rho_{Composite}=\rho_{isopropanol}\;\times \;\frac{m_{Composite\;in\;air}}{m_{Composite\;in\;air}-\;m_{Composite\;in\;isopropanol}}$$

The results were then compared to the theoretical densities calculated using the rule of mixtures. Furthermore, the true weight percentages of PGP were obtained by polymer burn-off at 650 °C for 1 h.

Gel permeation chromatography (GPC) was used to measure the PDLLA weight average molecular weight (*M*_w_), as-received and after the different processing stages. The PGPs were removed after dissolving the PDLLA in tetrahydrofuran using 0.22 µm filters. GPC (Viscotek-TDAMax, Malvern Panalytical Ltd., Malvern, UK) was conducted at 25 °C, using polystyrene (PS 99K) as reference.

#### 2.2.2. Scanning Electron Microscopy (SEM)

The cryo-fractured surfaces of the neat and composite monoliths and foams were examined using a field emission SEM (Hitachi S-4700, Hitachi America Ltd., Tarrytown, NY, USA). Samples were mounted on brass studs and coated using an Emitech K575X Peltier (Quorum Technologies Ltd., Lewes, UK) Cooled platinum coater (under argon) before being analyzed using a back-scattered electron mode with accelerating voltages of 2 kV.

#### 2.2.3. Computed X-ray Micro-Tomography (Micro-CT)

Micro-CT analysis was performed using a SkyScan 1172 (SkyScan, Kontich, Belgium), adapting a previously developed protocol [31]. In brief, freeze-cut samples (20 × 20 × 8 mm^3^) were analyzed with a resolution of 9.7 µm through a 360° flat-field corrected scan at 67 kV and 178 µA, and then reconstructed (NRecon software, SkyScan) with a beam hardening correction of 10, a ring artefact correction of 20 and an “auto” misalignment correction. The intensity of the CT scan 8-bit images generated was dependent on the density of air, PDLLA, and PGP. The 2D and 3D analyses of pore size, total porosity, and percentage of open pores (software CTAn, SkyScan) were carried out using a grayscale intensity range of 10 to 255 (8-bit images) in order to remove the background noise. The edges of the samples (2 mm thick for each dimension) were not considered in the analysis in order to avoid the artefacts intrinsic to the cutting procedure. Because of the differences in the densities of PDLLA and PG, it was possible to differentiate between the two materials using an intensity range of 10 to 30, and a threshold above 30, respectively. This criterion allowed the 3D reconstruction and the visualization of the different phases (CTVol software, Skyscan). PDLLA and PGP phases were pseudo-colored in grey and red, respectively.

#### 2.2.4. Mechanical Analysis

The effect of PGP content on the mechanical properties of the composite monoliths (nonporous) was investigated through measuring their flexural strength and Young’s modulus via three-point bending. Specimens (n = 3) were tested with a cross-head speed of 1 mm/min using a 1 kN load cell in accordance to ASTM D 790-95a:1996 (width-to-depth aspect ratio = 16) in an Instron mechanical testing instrument 5582 (Instron Ltd, Norwood, MA, USA).

Compression mechanical testing was also conducted to analyze the effect of PGP content on the compressive strength and Young’s modulus of the foams. Specimens (n = 3) were tested with a cross-head speed of 10% of the thickness/minute using a 1 kN load cell (Instron 5582, Norwood, MA, USA) in accordance to ASTM D 1621-00.

### 2.3. Statistical Analysis

Statistical analysis was performed to test the significance in difference between two mean values by using the Student’s *t*-test that was used to determine the *p*-values at a significance level of 0.05. Any statistically significant differences in the pore size, porosity, and open pore fraction of the foams, generated through micro-CT, were determined using one-way ANOVA with a Tukey-Kramer’s post-hoc multiple comparison of means. The level of statistical significance was set at *p* = 0.05.

## 3. Results

### 3.1. PDLLA-PGP Composite Monoliths (Nonporous)

#### 3.1.1. Physical Properties

The density of the composites increased with PGP content (Table 1). Both theoretical and experimental weight percentage values of the composites confirmed the amount of PGPs used to fabricate each composite system.

There was no significant difference between the Mw of PDLLA in various composites (Table 1). Compared to as-received PDLLA granules, there was a slight thermal degradation in all the specimens including the neat PDLLA. Nevertheless, the PDLLA matrix of the composites did not appear to be considerably affected by the PGP incorporation.

#### 3.1.2. Morphological Characterization

**Figure 1:**
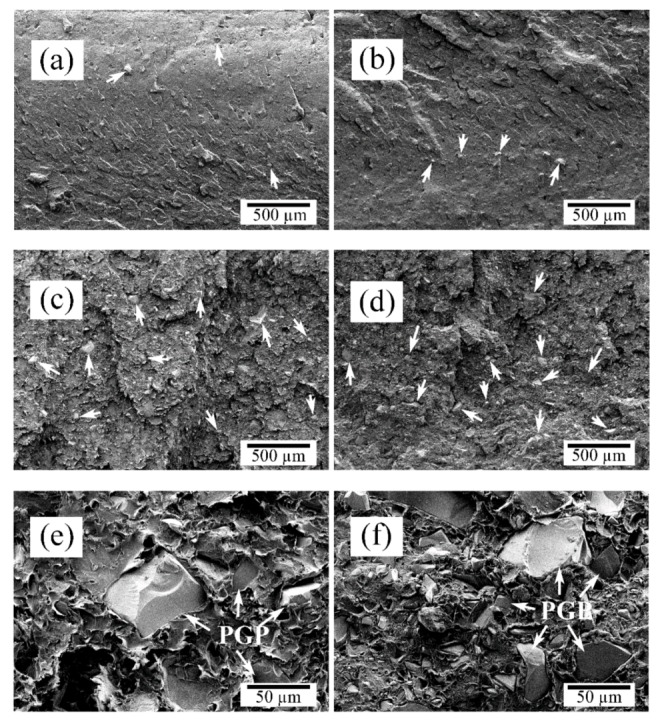
Scanning electron microscopy (SEM) micrographs of PDLLA-PGP composite monoliths (nonporous) with different PGP contents and magnifications: (**a**) PDLLA-5PGP (50×), (**b**) PDLLA-10PGP (50×), (**c**) PDLLA-20PGP (50×), (**d**) PDLLA-30PGP (50×), (**e**) PDLLA-20PGP (500×), and (**f**) PDLLA-30PGP (500×). The arrows indicate the glass particles (PGP).

SEM micrographs of as-processed composites confirmed the relatively uniform distribution of PGPs within the PDLLA matrix (Figure 1a–d). Higher magnification micrographs of PDLLA-20PGP and PDLLA-30PGP (Figure 1e,f, respectively) qualitatively demonstrated a good interfacial adhesion between the PGPs and the matrix.

#### 3.1.3. Mechanical Properties

**Figure 2:**
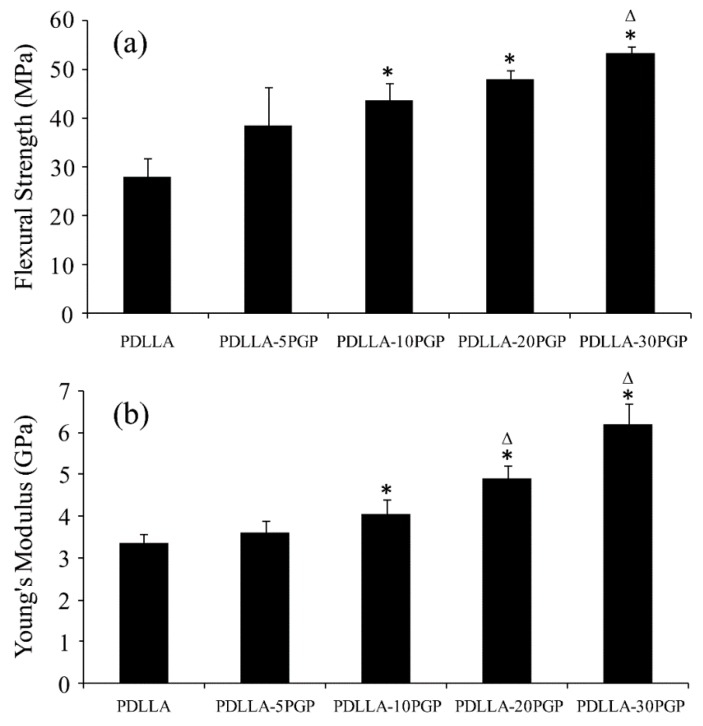
Mechanical properties of neat PDLLA and PDLLA-PGP composite monoliths (nonporous). (**a**) Flexural strength, (**b**) Young’s modulus. * Statistically significant compared to PDLLA (*p* < 0.05); Δ statistically significant compared to previous material (*p* < 0.05).

The flexural strength and modulus values of the PDLLA-PGP composite monoliths increased (*p* < 0.05) up to ~2-fold at 30 vol.% PGP when compared to those of the neat PDLLA (Figure 2a,b). The flexural strength of PDLLA-30PGP was significantly (*p* < 0.05) higher when compared to the other composites. PDLLA-20PGP and PDLLA-30PGP showed a statistically significant (*p* < 0.05) increase in Young’s modulus compared to other composites.

### 3.2. PDLLA-PGP Composite Foams

#### 3.2.1. Morphological Characterization

**Figure 3:**
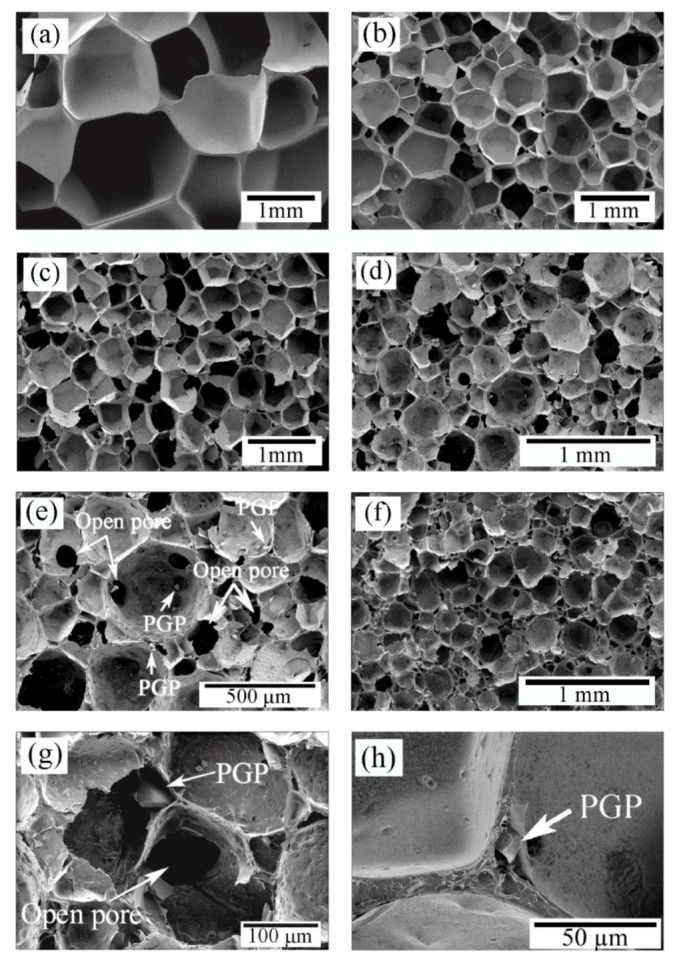
SEM micrographs of the neat and composite foams. (**a**) PDLLA, (**b**) PDLLA-5PGP, (**c**) PDLLA-10PGP, (**d**) PDLLA-20PGP, (**e**) PDLLA-20PGP at higher magnification, (**f**) PDLLA-30PGP, (**g**) PDLLA-30PGP at higher magnification, (**h**) PDLLA-PGP composite foam showing the presence of a PGP in the pore wall, creating pore wall rupture during foam expansion.

SEM micrographs of neat PDLLA and PDLLA-PGP composite foams are presented in Figure 3. The incorporation of 5 vol.% PGP resulted in a reduction in the pores size, which appeared to be dominated by closed pores, where PGPs were present within the pore walls. Increasing the amount of PGP to 10 vol.% resulted in further reduction of the pore size (Figure 3c). Well-distributed PGPs within the porous structures were observed in the SEM images (e.g., Figure 3e). While increasing the amount of PGP to 20 and 30 vol.% led to further pore size reduction (Figure 3d,f), there was a considerable increase in the number of open pores and resulting open windows (inter-pore openings) (Figure 3e,g). SEM observations revealed that the incorporation of PGPs was associated with open pores and resulting open windows. Also, the presence of a PGP in the pore wall resulting in disassociation at the matrix-particle interface, is shown in Figure 3h, as an example.

#### 3.2.2. Micro-CT

**Figure 4:**
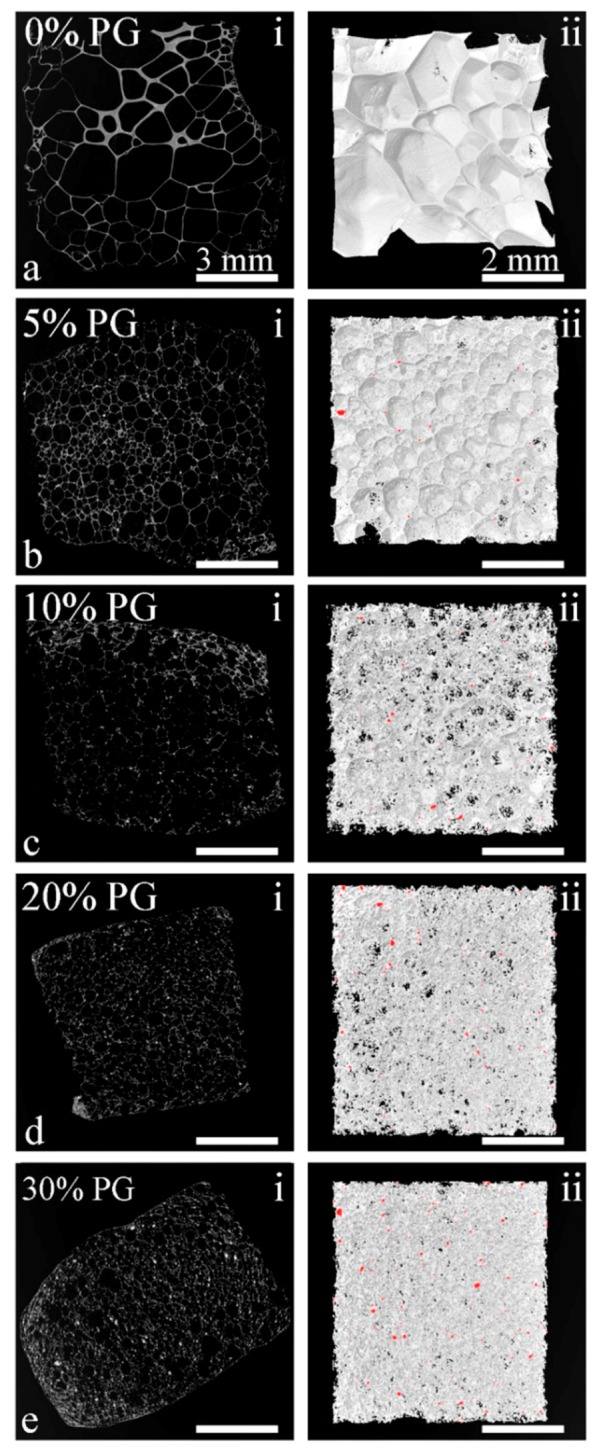
Micro-CT images of the SSF fabricated foams. (**a**) PDLLA, (**b**) PDLLA-5PGP, (**c**) PDLLA-10PGP, (**d**) PDLLA-20PGP, (**e**) PDLLA-30PGP. Note that, i and ii represent 2D and 3D images, respectively. The scale bars of the images on the left and right columns are 3 mm and 2 mm, respectively.

Micro-CT 2D and 3D images of the SSF fabricated composite foams indicated that PGP incorporation resulted in a reduction in pore size and increased open porosity in the microstructure of the PDLLA matrix (Figure 4 and Table 2) that were consistent with the SEM results. Pore size considerably decreased by increasing PGP content from an average of 920 µm for the neat PDLLA foam to 190 µm for PDLLA-30PGP. While there was a slight reduction in total porosity, an increase in PGP content led to a significant increase in the percentage of open pores, ranging from 7.7% for PDLLA-5PGP to 79% for PDLLA-30PGP (Table 2). In addition, there was a 2-, 6-, 14-, and 21-fold increase in the percentage of open pores for the composite foams with 5, 10, 20, and 30 vol.% PGP content, respectively. 

**Figure 5:**
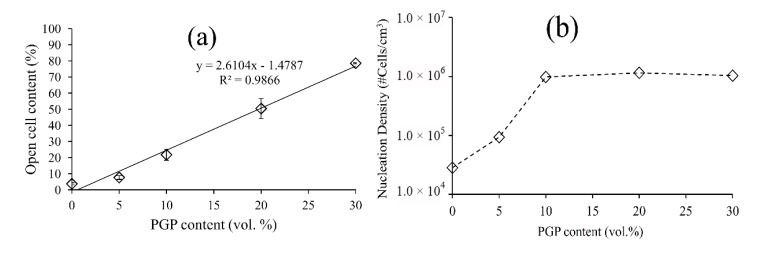
Open pore content and nucleation density of PDLLA-PGP composite foams. (**a**) Dependency of the percentage of open pores on PGP content, (**b**) nucleation density versus PGP content for PDLLA-PGP composite foams with different PGP content.

A linear correlation was observed between the content of open pores and the PGP content (Figure 5a). The nucleation density (β), defined by the number of pores per unit volume of the nonporous polymer, was evaluated using Equation (2) [32]:

(2) $$\beta \;≅\;\frac{6\;\times 10^{+12}\left[\left(\emptyset /100\right)/\left(1-\emptyset /100\right)\right]}{\pi d^{3}}$$

where *d* is the average diameter of the pores, and ϕ is the volume porosity. Increasing the PGP content impacted the nucleation density only at the lower volume fraction, typically less than 10 vol.%, as illustrated in Figure 5b. Above this value, the nucleation density was constant at approximately 1 × 10^6^ pores/cm^3^.

#### 3.2.3. Mechanical Properties

**Figure 6:**
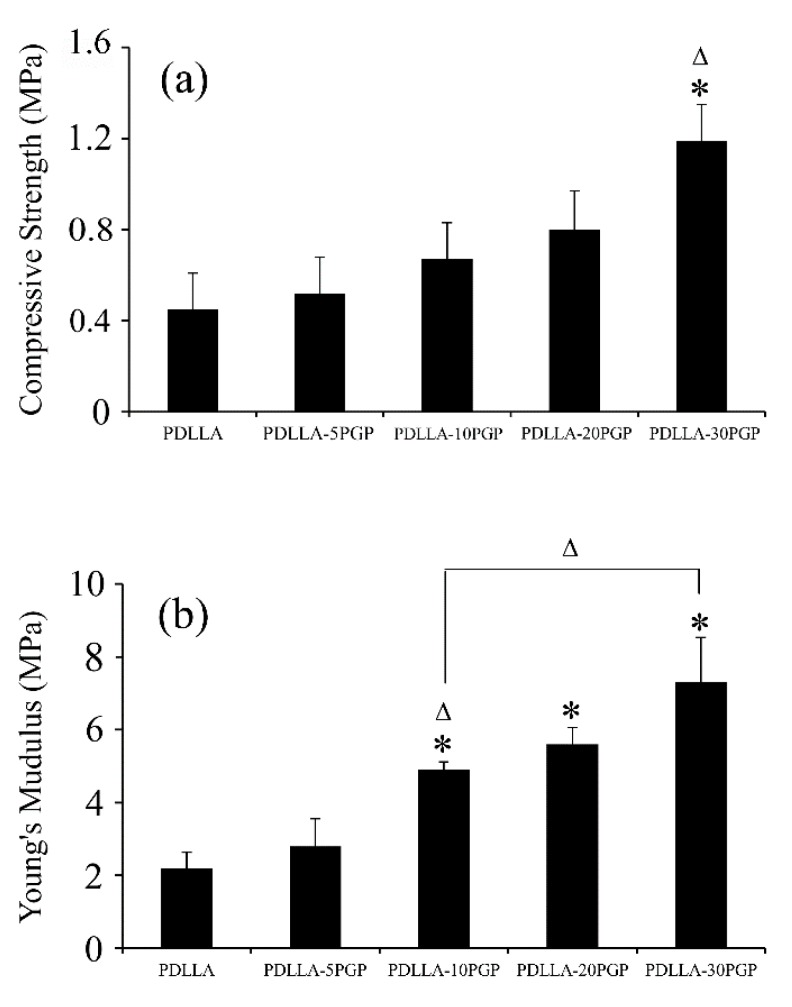
Mechanical properties of neat PDLLA and PDLLA-PGP composite foams. (**a**) Compressive strength, (**b**) Young’s modulus. * Statistically significant compared to PDLLA (*p* < 0.05); Δ statistically significant compared to previous material (*p* < 0.05).

The compressive strength and modulus of neat PDLLA and PDLLA-PGP composite foams are presented in Figure 6. There was a significant (*p* < 0.05) increase in the compressive strength of PDLLA-30PGP foams (Figure 6a) compared to that of the neat foam and other composite foams with lower PGP contents. A similar trend was observed with the modulus values where there was a statistically significant (*p* < 0.05) increase observed above 10 vol.% PGP incorporation (Figure 6b).

The Gibson and Ashby model, which has generally been used to model the modulus to the density/porosity of the foams [33], was applied to validate the modulus values by using Equation (3).

(3) $$\frac{E}{E_{0}}=C{\left(\frac{\rho }{\rho_{0}}\right)}^{n}=C\;{\left(1-P\right)}^{n}$$

where *E* and *ρ* are the modulus and density of the foam, respectively; *E*_0_ and *ρ*_0_ are the modulus and density of the nonporous materials, respectively; and *P* is the porosity of the foam (Table 1, Table 2 and Table 3). The constants C and *n* are dependent on the microstructure of the foam, where n generally has a value in the range 1 < *n* < 4 giving a wide range of *E*/*E*_0_ at a given density [34]. It has previously been confirmed that C equals 1 for the foams with dense struts [33]. The effect of incorporating PGPs into the PDLLA porous structure and the ability of Equation (3) to predict the mechanical anisotropy of the composite foams were assessed by calculating *n* values based on the experimental data while assuming C = 1 (Table 3).

It was found that *n* was within the predicted range for the Gibson and Ashby model (1 < *n* < 4). The value of n remained in the range 2 < *n* < 3 for 5, 10, and 20 vol.% PGP incorporation with slight variations and considerably increased to above 3 (*n* = 3.27) for the sample containing 30 vol.% PGPs.

## 4. Discussion

The composite fabrication technique used in this study resulted in well-dispersed PGPs within the PDLLA matrix, as indicated by arrows in Figure 1a–d. Previously, it was shown that SiO_2_-containing PG resulted in the degradation of polyester matrix leading to a significant reduction in the matrix molecular weight when processed at elevated temperatures [35,36]. This was attributed to the reactivity of surface Si–OH groups with the PDLLA ester bonds. In this study, the incorporation of TiO_2_-containing PGPs into PDLLA did not considerably change the molecular weight of the matrix (Table 1).

There was a statistically significant increase in the flexural strength of the nonporous composite monoliths compared to that of the neat PDLLA when the PGP content was ≥10 vol.% (Figure 2a). However, no statistical difference was observed between PDLLA-10PGP, PDLLA-20PGP, and PDLLA-30PGP. A statistically significant increase in Young’s modulus of the composites was also observed above 10 vol.% PGP (Figure 2b). There was an approximately two-fold increase in the flexural strength and modulus of the composites with 30 vol.% PGP content compared to those of the neat PDLLA. The strength and modulus of these composite monoliths were in the range of 38.5–53 MPa, and 3.6–6.2 GPa, respectively, and within ranges reported for trabecular bone [37,38,39]. 

After foaming, the neat PDLLA resulted in very large cellular structure and the incorporation of PGPs significantly decreased the pore size (Figure 3), yet still in the acceptable range of 200–900 µm for BTE application, as reported by Salgado et al. [4]. Although, it may not be possible to suggest an optimal pore size for the scaffolds because of the large number of bone features in vivo [40], larger pore size favors osteogenesis as it allows for sufficient nutrient supplies and the exchange of metabolic products. However, there is also an upper limit for the pore size due to the possible reduction in mechanical stability of the scaffolds and the surface area available for cell attachment. The presence of PGPs, especially at higher percentages, led to the creation of open windows and open pores (Figure 3e,g), which increased linearly with increasing PGP content (Figure 5a). In addition, since PGPs are soluble in aqueous environments [17,18], their dissolution in physiological fluids will further increase both porosity and open pore percentage.

The incorporation of PGPs into PDLLA reduced the pore size by promoting heterogeneous nucleation. Because of the increased pore nucleation sites, pore walls were thinner and more even, especially near the vortices (Figure 3h). Substantial vortices disappeared leaving pores with well-defined angular shape. Such morphology is usually associated with polymers exhibiting a strain hardening process [32], which suggests the occurrence of such behavior with the addition of the PGPs. There was a further reduction in pore size at 10 vol.% PGPs (Figure 3) with a predominant appearance in the surface texture and rupture of the pores walls, as indicated by the presence of spherical holes. This pore opening behavior was further magnified at 20 vol.% PGPs (Figure 3e), with holes present within most of the pores. In addition, the cross-section of the walls exhibited a porosity that was linked to the loss of adhesion between the PGPs and the PDLLA matrix because of the stress concentration at these locations as well as the further stretching of the polymer films creating such voids [41]. Although the morphological characterization of the composite monoliths (Figure 1) suggested a good interfacial adhesion between the matrix and PGPs (also validated through mechanical testing), disassociation at the matrix-particle interface in the porous structures (Figure 3) should have occurred during the biaxial stretching of the pore walls (Figure 3h). The presence of large glass particulates can weaken the pore walls during expansion leading to higher probability of such pore wall rupture. Larger PGPs having greater diameters than the pore wall thickness (10–20 µm) are more prone to cause pore wall rupture and hence pore opening. However, for particulates much smaller than the wall thickness, voids are formed during stretching as a result of local stress concentrations, locally weakening the film and contributing to the complete rupture of the pore wall. 

Although small addition of glass particulates deemed to contribute to a more uniform deformation throughout the polymer via a strain hardening mechanism, increasing the PGPs content above a certain limit (approximately between 5 and 10 vol.%) had the opposite effect, inducing stress concentrations that eventually led to necking and further pore wall rupture and opening. Increasing the glass content further to 30 vol.% had a strong effect on the opening of the pores (Figure 3g). However, this increase in open pores may have resulted in gas loss during the foaming process, which limited the expansion of the cellular structure. With approximately the same nucleation density as for the composite with 20 vol.% PGPs, the total porosity remained below 80% with an average pore diameter of less than 200 µm (Table 2). 

The 30 vol.% PGPs incorporation led to ~2.6-fold increase in the compressive strength of the foams compared to that of the neat PDLLA foam (Figure 6). The Young’s modulus of the foams noticeably increased at 10 vol.% PGP incorporation followed by ~3.3-fold increase at 30 vol.% PGP content. Pore diameter variations, which decreased with glass content, stabilizing at approximately 200 µm may provide an explanation to the significant increase in Young’s modulus at 10 vol.% content. It is expected that the glass incorporation would have a similar effect on the modulus of both nonporous and foamed composites which can be seen in our results (Figure 2b and Figure 6b). Although the impact of the pore diameter on the mechanical properties of highly porous bone scaffolds has been the subject of numerous studies and is not yet fully understood [42], the results of this study indicated two- and three-fold increases in the moduli of the 30 vol.% PGP composite monolith and foam, respectively, when compared to those of the corresponding neat PDLLA structures. 

The Gibson and Ashby model was applied to the experimental data, in which the n values of the composite foams (Table 3) were in the range reported for this model confirming its suitability in predicting the behavior of these composites at any filler content. In addition, the constant n, which depends on the foam microstructure, obtained in this study was comparable to those previously reported for thermally induced phase separation (TIPS) generated PLGA-(0, 1.5, and 6.7 vol.%)TiO_2_ [43] and PDLLA-(0, 2, and 15 vol.%) Bioglass^®^ [12]. This highlights the fact that, *n* is a microstructure-dependent constant and is not affected by the type or amount of the filler. Blaker et al. [12] incorporated up to 15 vol.% Bioglass^®^ into PDLLA foams and stated that *n* remained almost in the same range 2 < *n* < 3. In our study, while the value of *n* also remained in the range 2 < *n* < 3 for 5, 10, and 20 vol.% PGP with similar values compared to the ones reported by Blaker et al. [12], *n* increased to above 3 for the foam with 30 vol.% PGPs content (Table 3). This change in mechanical properties may be associated with changes in the foam morphological properties, i.e., reduction in pore size and porosity, and the increase in the open pore content (Table 2 and Figure 5a).

The results obtained from the SSF technique appear to be more attractive than those from batch foaming based on the rapid depressurization in the polymer molten state followed by the cooling of the cellular structure. Adopting this technique, Georgiou et al. [28] developed composite foams based on commercial bioresorbable semi-crystalline PLA and a PG formulation. Although up to 20 wt.% PGP content was investigated, foams with the porosity >75% could only be achieved for 5 and 10 wt.% PGP. In contrast, SSF combined with using amorphous PDLLA allowed for the production of foams with higher PGP content, up to 30 vol.% (~46 wt.%), and increased porosity and open pore structures. Salerno et al. [44], produced poly(ε-caprolactone) (PCL)/hydroxyapatite (HA) nanocomposite foams with porosities up to ~90% via a two-step depressurization solid-state supercritical CO_2_ (scCO_2_) foaming technique. They showed that the depressurization profile has a significant impact on the pore structure of the scaffolds such that at the intermediate pressure of 10 MPa, composite scaffolds with bimodal pore size distributions consisting of macro-porosity and micro-porosity with mean pore sizes in the range of 100–300 µm and 50–70 µm, respectively, were produced [44]. In another study by the same group [45], scCO_2_ foaming and porogen leaching techniques were combined to develop BTE scaffolds of PCL/thermoplastic zein (TZ) containing 20 wt.% HA particles. The PCL/TZ-HA composite scaffolds showed a relatively low porosity of ~63% on average with a bimodal pore structure consisting of a macro-porosity of mean pore size of 121 ± 49 µm and a micro-porosity in the range of 1–10 µm. Similar to our observations, it was found that by adding the HA particles, both porosity and pore size of the foams decreased [45]. Mathieu et al. [46] used scCO_2_ foaming to produce PLA/(1–30 wt.%)HA nanocomposite foams with a wide range of characteristics. It was shown that the PLA/HA foams exhibited a more heterogeneous structure because of the tendency of the HA particles to agglomerate. Composite foams exhibited higher average pore diameter with more closed pores (not suitable for BTE) compared to the neat PLA foams, which are in contrast with our results. It was argued that the higher matrix viscosity because of the presence of inorganic particles led to more closed pores [46]. The study also reported that, increasing the filler content decreased the porosity and increased the density and the compressive modulus of the foams [46], similar to the results of our study. Also, our results indicated that the SSF technique can offer more advantages and flexibility to design composite foams (e.g., bone scaffolds) with various morphological, physical, and mechanical properties compared to other foaming methods such as salt-leaching and TIPS. Navarro et al. [47] produced composite scaffolds of poly(95L/5DL) lactic acid reinforced with PGPs (44.5P_2_O_5_-44.5CaO-6Na_2_O-5TiO_2_) using salt-leaching technique (94% *w*/*w* NaCl particles). Although high levels of porosity (up to 97%) were achieved, the compressive mechanical properties were in the kPa range. Blaker et al. [48] used TIPS technique to produce porous microstructures composed of PLGA and silver-doped PGs. Although TIPS process results in high porosity and interconnectivity, the pore size is normally smaller than that proposed as optimal for BTE (200–900 µm [4]). In that study, porous macro-spheres (up to 2000 µm in diameter) with pores in the range of 30–70 µm were produced [48]. In another study, PDLLA-Bioglass^®^ composite foams fabricated by TIPS process resulted in tubular pores of ~100 µm [12]. 

In this study, the incorporation of glass particulate was found to be highly beneficial to the SSF process applied to PDLLA as it synergistically contributed to the induction of a stable expansion and the formation of foams with improved mechanical properties and high porosities and pore interconnectivity; all of which are desirable for designing bone scaffolds. Overall, the addition of the glass particulates to PDLLA promoted nucleation resulting in smaller pores with thinner walls. Larger particulates (>20 µm) created the pores during late pore growth, and the smaller particulates (<20 µm) modified the rheological behavior [46] (increased melt strength) such that homogeneous deformations occurred throughout the cellular structure under expansion, at least in its early stage. Thus, despite its limitations (e.g., a relatively long foaming process, dependency on specialized equipment such as high-pressure autoclave, and not being applicable to all types of polymers), SSF appears to be a promising technique for producing highly porous composite foams for BTE applications.

## 5. Conclusions

PDLLA-PGP composite monoliths and foams were successfully produced with up to 30 vol.% PGP content via melt extrusion and compression molding followed by solid-state foaming using CO_2_. This foaming technique allowed for the incorporation of a broad range of PGP volume fraction, which can be very beneficial in BTE applications, since various particle contents may be required depending on their composition and the target application. This foaming process was shown to be an effective route for producing macro-porous structures in PDLLA-PGP composites, generating approximately 79 to 91% porosity. Although the pore size decreased with increasing the PGPs content, it yet remained in the proposed acceptable range for bone tissue engineering applications (200–900 µm). In addition, the percentage of open pores (i.e., interconnectivity) beneficially increased by increasing the PGPs content (up to 78% at 30 vol.% PGP). It was hypothesized that the presence of PGPs led to the creation of open windows during the foam expansion, which opened up the pores. The incorporation of PGP also increased the compressive strength and modulus of both nonporous monoliths and foams. The modulus-porosity relationship of the foams was confirmed by the Gibson and Ashby model, and effective microstructural changes were shown to occur by 30 vol.% PGP incorporation. Although further in vitro studies are required to assess the characteristics of these composite foams (scaffolds). In summary, the PDLLA-PGP foams produced in this study are promising in terms of morphological, physical, and mechanical properties for potential applications in bone tissue engineering.

## Figures and Tables

**Table 1:** Material codes, phosphate-based glass particulate (PGP) content, density of composites, molecular weight (Mw) of the PDLLA matrix after processing, and relative Mw of the PDLLA matrix of composites to the neat PDLLA after processing.

Material Code	PGP (vol.%)	PGP Content Experimental (wt.%)	Density (g.cm^−3^)	PDLLA Molecular Weight (*M*_w_: Daltons)	Relative *M*_w_
PDLLA	-	-	1.26	51,594 ± 131	1.000
PDLLA-5PGP	5	9.8 ± 0.65	1.27 ± 0.017	51,133 ± 149	0.991
PDLLA-10PGP	10	16.6 ± 0.09	1.35 ± 0.016	50,954 ± 91	0.988
PDLLA-20PGP	20	32.9 ± 0.08	1.51 ± 0.017	49,936 ± 13	0.968
PDLLA-30PGP	30	45.9 ± 0.14	1.63 ± 0.02	51,445 ± 211	0.997

**Table 2:** Pore size, total porosity, and percentage of open pores for PDLLA, and PDLLA-PGP composite foams measured by micro-CT.

Material Code	Pore Size (µm)	Total Porosity (vol.%)	Percentage of Open Pores
PDLLA	920 ± 640	92 ± 1.59	3.7 ± 0.65
PDLLA-5PGP	530 ± 230	87.9 ± 0.44	7.7 ± 1.17
PDLLA-10PGP	270 ± 210	91 ± 0.51	22 ± 3.47
PDLLA-20PGP	230 ± 140	88 ± 0.48	50.4 ± 6.24
PDLLA-30PGP	190 ± 130	78.8 ± 0.35	78.6 ± 0.35

**Table 3:** Modulus of the nonporous (*E*_0_) and porous (*E*) composites as well as *n* values obtained from the Gibson and Ashby model (Equation (3)).

Material Code	*E* (MPa)	*E*_0_ (GPa)	*n*
PDLLA	2.22 ± 0.24	3.36 ± 0.22	2.68
PDLLA-5PGP	2.79 ± 0.40	3.62 ± 0.28	2.49
PDLLA-10PGP	4.89 ± 0.14	4.05 ± 0.33	2.31
PDLLA-20PGP	5.65 ± 0.22	4.91 ± 0.31	2.67
PDLLA-30PGP	7.27 ± 0.65	6.19 ± 0.45	3.27

## Supplementary Materials

The following are available online at https://www.mdpi.com/2073-4360/12/1/231/s1, Table S1: CO_2_ solubility in PDLLA at different pressures and times at room temperature.
