# Supplementary material for: Poly(d,l-Lactic acid) Composite Foams Containing Phosphate Glass Particles Produced via Solid-State Foaming Using CO2 for Bone Tissue Engineering Applications
Source: Polymers (Basel). 2020 Jan 17;12(1):231. doi: 10.3390/polym12010231 (PMC7023552; doi:10.3390/polym12010231)
Supplement: Supplementary file 1 [file polymers-12-00231-s001.pdf]

Supplementary Material

Poly(D.L-Lactic acid) Composite Foams Containing Phosphate Glass Particles Produced via Solid-State Foaming Using CO_2 for Bone Tissue Engineering Applications

Maziar Shah Mohammadi [1,†], Ehsan Rezabeigi [1], Jason Bertram [1,‡], Benedetto Marelli [1,§], Richard Gendron [2], Showan N Nazhat [1,*], Martin N Bureau [1,2,¥]

[1] Department of Mining and Materials Engineering, McGill University, Montreal, QC, H3A 0C5, Canada

[2] National Research Council Canada, Boucherville, QC, J4B 6Y4, Canada

[†] *Now at* Department of Physical Medicine and Rehabilitation, USUHS, USA

[‡] *Now at* Air Canada, QC, Canada

[§] *Now at* Department of Civil and Environmental Engineering, Massachusetts Institute of Technology, Cambridge, MA, USA

[¥] *Now at* Sanexen Environmental Service Inc., QC, Canada

* Correspondence: showan.nazhat@mcgill.ca

Supplementary Table S1. CO_2 solubility in PDLLA at different pressures and times at room temperature. Diffusion of CO_2 within the polymer is time-dependent and a homogeneous concentration may be only reached after a given saturation period, which is a function of the sample geometry (thickness) and pressure. At pressures below 2 MPa, CO_2 content was not more than 3 wt.% which led to an ineffective foaming. For example, CO_2 content was 2.76 wt.% at 1.7 MPa after 164 h. At higher pressures (*e.g.,* 3.4 MPa), even at shorter soaking times (*e.g.,* 27 h), the samples were soft and sticky, and bubbles formed within them as soon as they were removed from the autoclave to atmospheric pressure. This indicates that the resulting T_g was below room temperature. Knowing that 6.1 wt.% of CO_2 was dissolved into the PDLLA sample suggests a plasticization level of ~ 6.5 ºC/wt.% of CO_2. Moderate pressures (~ 2.4 MPa) were found to be appropriate to obtain sufficient dissolved CO_2 (~ 5 wt.%) for effective foaming, leading to uniform expansion and high porosity. However, at least 72 h was required for a complete CO_2 saturation of the samples. Note that, the soaking time is related to the diffusivity of the gas and the thickness of the sample. Diffusivity is slow below T_g of the polymer and increases rapidly in the rubbery state (above T_g). This explains the fast dissolution at high pressures such as 3.4 MPa; however, very long soaking times were required at moderate pressure such that the sample remained in the glassy state.

Pressure (MPa)	Exposure Time (h)	CO_2 content (wt.%)	Note
1.7	21, 96, 164	1.06, 2.03, 2.76	
2.2	22	2.91	Few bubbles present inside the sample
2.4	72	5.48	Selected for the foaming experiments
3.4	27	6.1	Plasticized at room temperature
